# Socio-Demographic and Health Data of Adolescents Identifying as Transgender or Gender Diverse in the Born in Bradford’s Age of Wonder Cohort.

**DOI:** 10.12688/wellcomeopenres.24147.2

**Published:** 2026-02-20

**Authors:** Jake Camp, Natalie Kashirsky, Sofia Pinckard, Sadie Hambleton, Abigail Oliver, Lindsay Smith, Ellena Badrick, Sunil S Bhopal, Julian Baudinet, Michael Absoud

**Affiliations:** 1 Department of Psychology; School of Mental Health and Psychological Sciences; Institute of Psychiatry, Psychology, & Neuroscience, King's College London Department of Psychology, London, England, SE5 8AF, UK; 2 The NHS Children & Young People's Gender Service (London), London, UK; 3 Bradford Teaching Hospitals NHS Foundation Trust, Bradford Institute for Health Research, Bradford, England, BD9 6RJ, UK; 4 London School of Hygiene and Tropical Medicine Faculty of Epidemiology and Population Health, London, England, UK; 5 Institute of Psychiatry, Psychology, & Neuroscience, King's College London Department of Psychological Medicine, London, England, SE5 8AF, UK; 6 King's College London Department of Women & Children's Health, London, England, UK

**Keywords:** Transgender; Gender Diverse; Adolescents; Mental Health; Health; Socio-Demographics

## Abstract

Background The number of adolescents identifying as Transgender and Gender Diverse (TGD) are thought to be growing. However, robust data detailing the rates for self-identification is lacking for those under 16 years of age. Moreover, there are few studies which have characterised the socio-demographic and health information of this community to inform service planning. The current study reports the proportion of 12 to 15 years olds within an ethnically diverse population cohort dataset who were classified as TGD, as well as their socio-demographic and health profile. Methods A cross-sectional observational study was conducted using the Born in Bradford, Age of Wonder cohort dataset. This includes 4,954 adolescents from 15 schools in the Bradford District (UK). Data were provided on adolescents’ assigned sex at birth and gender identity, other socio-demographic variables, and health proxies (e.g. anxiety and depression symptoms) which were stratified by those who were categorised as TGD and those as cisgender. Results Of the sample, 1.40% identified as TGD. There was a higher frequency of individuals identifying as a transgender boy (female assigned sex; 0.50%) and nonbinary (female assigned sex; 0.50%) compared to transgender girls (male assigned sex; 0.20%) and nonbinary (male assigned sex; 0.20%). The TGD group were more likely to be from a White ethnicity background, have no practiced religion, have access to free school meals, have special educational needs (SEN), and self-report the presence of physical or mental illness. TGD young people also scored significantly higher on anxiety and depression symptoms. There were no significant differences between groups in age indices, country of birth, or national deprivation quartiles. Conclusions This study provides an insight into the socio-demographic composition of the TGD group compared to cisgender group, within this population cohort dataset. This study also suggests increased health inequalities within TGD populations compared to cisgender populations.

## Plain Language Summary

**Who this research was conducted by:**

This research was conducted by researchers at King’s College London, the NHS Children and Young People’s Gender Service (London), and the Bradford Institute for Health. This study used data that was collected as part of the Born in Bradford data collection. Specifically, this was data collected from people aged 12 to 15 years old from schools across the Bradford (UK) district.

**What we were interested in:**

We were interested in how many young people identified as transgender or gender diverse (e.g. non-binary, gender queer), and who were more likely to identify as transgender or gender diverse based on other characteristics like ethnicity and sex assigned at birth. We were also interested in whether transgender and gender diverse young people had more health concerns compared to those who were not transgender or gender diverse (i.e. cisgender young people).

**What we found:**

We found that 1.40% of young people identified as transgender or gender diverse, and these were more likely to be people who were assigned female sex at birth compared to males. Young people from White ethnicity backgrounds, who did not practice a religion, had access to free school meals, and had special educational needs were more likely to identify as transgender and gender diverse. Finally, transgender and gender diverse young people reported higher physical and mental health concerns compared to cisgender young people.

**What this means:**

We think that this provides an insight into who is more likely to report a transgender or gender diverse identity and their health needs. This is important as it helps us understand who may need more support. The findings may be limited because some schools did not take part in the study and so the findings do not represent the whole population.

## Introduction

Transgender and other Gender Diverse (TGD) people are individuals whose gender identity does not exclusively match with their sex assigned at birth, inclusive of non-binary and binary transgender individuals ( Corbett *et al.*, 2024). Gender identity usually begins to develop around the age of three years, often showing fluidity in early childhood before stabilising during puberty, although variation is common ( Corbett *et al.*, 2024). Recording of gender diversity in those under 18 years of age, within the UK, has increased over 50-fold from 2011 to 2021 in primary care settings ( Jarvis *et al.*, 2025). In the UK Census 2021 ( Office of National Statistics [ONS], 2021), one in 200 people of those aged 16 and over reported a TGD identity ( ONS, 2023a) or 1.10% in UK population cohort studies ( White *et al.*, 2023). This rising proportion of TGD identification has also been characterised by an increase in the ratio of female assigned sex at birth people visiting gender services and identifying as TGD, compared to male assigned sex people ( Taylor *et al.*, 2024), as well as being identified in non-clinical studies ( Chew *et al.*, 2020). TGD prevalence data for those younger than 16 years of age is currently lacking within UK population cohort studies. However, such studies from elsewhere suggest around 1.20% to 8.40% of children and adolescents are TGD, depending on the definition and measurement methods ( Chew *et al.*, 2020; Zhang *et al.*, 2020).

Increases in the prevalence of recorded TGD identities within the UK may be due to greater availability of information and support, increased societal and clinical awareness, and improved acceptance leading to a partial destigmatisation ( Zhang *et al.*, 2020). However, data from population cohorts is needed to better understand whether these apparent increases are reflected in the population as a whole.

TGD youth also experience significant health inequalities in physical and mental health domains compared to their cisgender peers ( Black *et al.*, 2023; Chew *et al.*, 2020; Klinger *et al.*, 2024). Research suggests that these are due to multiple complex factors including social prejudice, discrimination, barriers to accessing support, and systemic inequities ( Drabish & Theeke, 2022; Watkinson *et al.*, 2024). There are several factors also associated with health disparities, which are likely helpful for aiding the understanding of what risk factors contribute to these inequalities. For example, TGD children and young people are more likely to come from a lower socio-demographic background compared to their cisgender peers ( Carpenter *et al.*, 2020), which is often associated with poorer health outcomes ( Quon & McGrath, 2014). This highlights the importance of measuring the socio-demographics of TGD youth populations to understand potential intersectional vulnerabilities and needs.

TGD youth from minoritised ethnicity and race-related backgrounds in the United States of America have also been found to experience higher rates of mental health symptoms compared with their cisgender peers ( Vance *et al.*, 2021). Little is known about UK-based TGD children and young people within minoritised ethnicity and religious communities, as research with TGD populations has been being predominantly conducted with majority ethnically White groups ( Millar & Brooks, 2022).

Given that most UK research has focused on individuals aged 16 and older, and is often focused on White and clinical populations, there is a need to further understand the proportion, socio-demographic, and health profile of younger adolescents who identify as TGD in the general population. Even in studies that have looked at TGD youth aged 12 to 15 years, these researchers did not disaggregate sociodemographic variables by TGD and cisgender groups ( Black *et al.*, 2023). The current study aims to describe the proportion of TGD adolescents within the ethnically-diverse Bradford district (UK), via a cross-sectional dataset which is part of a larger cohort study ( Shire *et al.*, 2024). The second aim is to understand if there are differences in socio-demographic and health profile of this group, compared to cisgender young people. The research questions are as follows:

1. What is the proportion of adolescents in the Bradford population cohort that identify as a gender identity different from their assigned sex at birth (TGD), compared to those who identify with a gender identity congruent with their assigned sex (cisgender)?
2. Are there socio-demographic differences between the TGD group and the cisgender group?
3. Is there a difference in self-report physical and mental health, and depression and anxiety disorder symptom scores, between TGD groups and cisgender groups?

## Methods

### Ethical considerations

The Born in Bradford’s Age of Wonder Cohort methodology was provided ethical approval by the Bradford Leeds NHS Research Ethics Committee (Ref: 21/YH/0261; ethical approved provided on 22/12/2021), which ensured that this study adhered to the Declaration of Helsinki. Parents/carers of adolescent participants were recruited on an informed opt-out basis and those adolescents whose parents did not opt out provided written assent ( Shire *et al.*, 2024). This research was a secondary data analysis conducted using data from the Age of Wonder dataset provided by the Born in Bradford (BiB) project. Prior to accessing the data, the research team from King’s College London (KCL) signed a data sharing agreement with BiB, ensuring compliance with ethical and legal standards which included provisions for data confidentiality, participant privacy, and the responsible use of data.

### Design

This cross-sectional analysis of an observational cohort study explored the proportion of an adolescent population who identified as TGD within the Born in Bradford, Age of Wonder dataset. This is part of a continuation and expansion of the Born in Bradford, longitudinal birth cohort study based in Bradford, UK ( Shire *et al.*, 2024). This study examined socio-demographic and health profiles of TGD youth compared to cisgender youth.

### Sample

**Figure 1.**
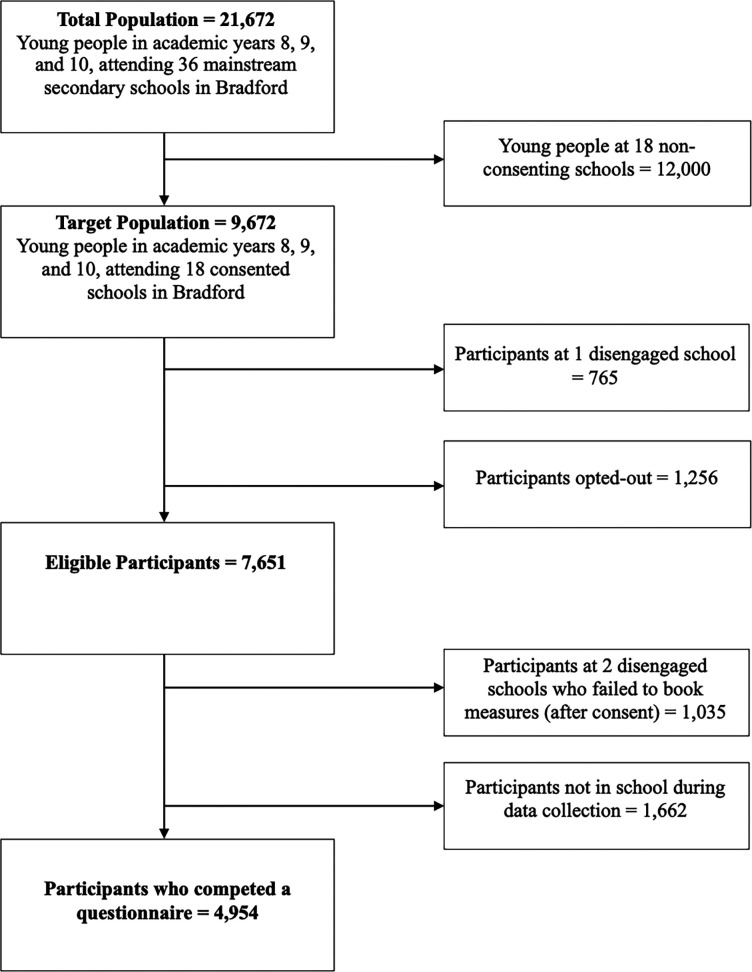
Sample Recruitment and Attrition.

Within the Age of Wonder dataset there were 4,954 participants aged 12 to 15 years (see Figure 1 for sample attrition). Mainstream schools within the Bradford District were approached to participate in the data collection. Participants were eligible to participate if, at the time of recruitment, they were: attending a school within the Bradford District that consented to take part; were in school years 8 to 10; the young person assented to take part; and that the schools were able to contact parents/carers with information about the study and then did not opt out. Participants were not eligible to take part if the parent/carer opted out, the participant did not assent, or a teacher deemed it inappropriate for them to participate.

This figure provides information about how many participants were recruited and sample attrition for the Age of Wonder cohort data collection.

### Procedures

An in-depth description of the procedures employed to collect the data are described in Shire *et al.* (2024). Online questionnaires were developed in consultation with experts, parents, and young people, and were completed via the REDCap online survey platform hosted by Bradford Teaching Hospital NHS Foundation Trust ( Harris *et al.*, 2019). Questionnaires were administered by school staff by sending a unique link to students’ email address or via tablet computers supervised by members of the research team. Schools also provided data from their records (more detail in the Measures section). Data was collected between September 2022 and July 2023.

### Measures

Of the data utilised in this study, schools provided data on the participants’ age and school year group, ethnicity, whether the participant had access to free school meals in that academic year, and their Special Educational Needs (SEN) status based on their records. A self-report questionnaire, with multiple choice and free text response options, asked about their sex (Question: “What is your sex? [The biological sex on your birth certificate]. A question about gender identity will follow in the survey”; response options: “ *female*”, “ *male*”, “ *do not wish to answer*”, and “ *wish to enter own response*”, which had space to self-describe) and gender identity (Question: “What is your gender?”; response options: “ *female*”, “ *male*”, “ *non-binary *”, “ *wish to enter own response*” with space to self-describe, and “ *do not wish to answer*”); alongside whether the participant considered themselves to have a religion (“ *yes*” or “ *no*”), their country of birth (200 drop-down options), and whether they have any physical or mental health conditions/illnesses (“yes” or “no”; Shire *et al.*, 2024). National deprivation deciles were determined by comparing the participants’ postcodes against the national deprivation decile postcodes ( Ministry of Housing, Communities and Local Government, 2019).

Symptoms of anxiety and depression were measured via the self-report 25-item Revised Children’s Anxiety and Depression Scale (RCADS-25; Ebesutani *et al.*, 2012). This scale included 25 items measuring anxiety and depression symptoms on a scale rated from “ *Never*” (0) to “ *Always*” (3). Raw scores are calculated for anxiety and for depression and then are scaled based on age and sex to derive a t score ( Cundill Centre for Child and Youth Depression, 2021). Higher scores indicate higher symptom severity. Scores below 65 are considered within the “normal” range; scores ranging from 65 to 69 are considered “borderline clinical”; and scores above 70 are in the “clinical” range ( Cundill Centre for Child and Youth Depression, 2021). The RCADS-25 has demonstrated good validity and reliability ( Ebesutani *et al.*, 2012; Klaufus *et al.*, 2020; Piqueras *et al.*, 2017).

### Data analysis

Data were analysed in SPSS (V29). Data were presented as descriptive statistics and differences in proportions of categorical variables were analysed using Chi Square analyses. Variables were grouped so that the number of participants in any group was *N = ≥5.* This was done to prevent the possibility of re-identification via specific socio-demographic variable combinations. Those who opted to not describe their sex ( *N* = 81) or gender ( *N* = 38) were treated as missing data. Gender identity data were compared against sex assigned at birth to determine the number of those who would be categorised as cisgender (assigned sex and gender identity matched) and those who would be categorised as transgender or gender diverse (assigned sex was different from gender identity). Self-described gender identities were merged into these categories where possible ( *N* = 15) and treated as missing data where not possible ( *N* = 16). The ethnicity groups were collapsed to also retain *N* = ≥5. These were aggregated into a Pakistani group, as the second largest ethnic group in the geographical area ( Shire *et al.*, 2024) those from White groups; and those from the remaining groups (see Table 1 for a further breakdown of the remaining groups).

**Table 1.** Socio-demographic and Health Data for the Cisgender and TGD Groups ( *N* = 4,954)

	Cisgender	TGD	Missing: Gender Not Reported	Missing Generally	*X ^2^*
Age (in Years)					
12	680 (98.40%)	11 (1.59%)	8 (0.92%)	171 (19.66%)	2.01
13	1,400 (98.59%)	20 (1.41%)	15 (0.89%)	258 (15.24%)	
14	1,444 (98.84%)	17 (1.16%)	17 (1.08%)	246 (15.70%)	
15	549 (98.04%)	11 (1.96%)	15 (2.25%)	91 (13.66%)	
Missing (uncategorised)				1	
School Year					
8	1,257 (98.36%)	21 (1.64%)	16 (1.02%)	276 (17.58%)	1.09
9	1,498 (98.81%)	18 (1.19%)	13 (0.73%)	242 (13.67%)	
10	1,318 (98.51%)	20 (1.49%)	26 (1.61%)	249 (15.44%)	
Missing (uncategorised)				0	
Sex on Birth Certificate					
Female	2,198 (98.21%)	40 (1.79%)	21 (0.92%)	14 (0.62%)	5.94*
Male	1,874 (99.10%)	17 (0.90%)	3 (0.16%)	17 (0.89%)	
Missing (uncategorised)				770	
Ethnicity Reported by School					
Asian, Black, and Mixed Groups ^a^	689 (99.14%)	6 (0.86%)	8 (0.94%)	150 (17.59%)	47.25**
Pakistani	2,110 (99.20%)	17 (0.80%)	26 (1.00%)	449 (17.26%)	
White ^b^	798 (95.80%)	35 (4.20%)	6 (0.64%)	106 (11.22%)	
Missing (uncategorised)				545	
Country of Birth					
Inside of the UK	3,377 (98.51%)	51 (1.49%)	42 (1.18%)	95 (2.67%)	0.89
Outside of the UK	596 (99.00%)	6 (1.00%)	11 (1.69%)	38 (5.84%)	
Missing (uncategorised)				738	
Religion					
Yes, Has Religion	3,369 (99.38%)	21 (0.62%)	36 (1.02%)	110 (3.11%)	79.93**
No Religion	682 (95.12%)	35 (4.88%)	18 (2.37%)	26 (3.42%)	
Missing (uncategorised)				657	
Free School Meals					
No	2,869 (98.80%)	35 (1.20%)	42 (1.22%)	495 (14.39%)	4.60*
Yes	1,117 (97.90%)	24 (2.10%)	11 (0.78%)	268 (18.87%)	
Missing (uncategorised)				93	
National Deprivation Quartiles					
1 (poorest)	1,987 (98.51%)	30 (1.49%)	28 (1.13%)	431 (17.41%)	1.09
2	617 (98.56%)	9 (1.44%)	7 (0.94%)	116 (15.49%)	
3	540 (98.72%)	7 (1.28%)	5 (0.74%)	121 (17.98%)	
4 (richest)	595 (98.02%)	12 (1.98%)	12 (1.77%)	58 (8.57%)	
Missing (uncategorised)				379	
Special Educational Needs					
No	2,851 (98.82%)	34 (1.18%)	36 (1.05%)	509 (14.84%)	8.56**
Yes	517 (97.18%)	15 (2.82%)	10 (1.73%)	128 (22.11%)	
Missing (uncategorised)				854	
Physical or Mental Illness					
Yes	503 (94.91%)	27 (5.09%)	14 (2.43%)	33 (5.72%)	63.28**
No	3,481 (99.20%)	28 (0.80%)	39 (1.07%)	85 (2.34%)	
Missing (uncategorised)				744	

Note. % for TGD and cisgender young people represent the percentage for the available data, whereas % for those missing by gender not being reported and missing for other reasons represent the whole sample. TGD = transgender and gender diverse. ASAB = assigned sex at birth.

^a^ This group is made up of ethnicities including African (11.10%), Any other Asian background (9.30%), Any other Black background (2.20%), Any other ethnic group (8.70%), Any other Mixed ethnicity (9.30%), Arab (1.60%), Bangladeshi (24.00%), Caribbean (1.80%), Chinese (1.20%), Indian (16.80%), White and Asian (10.30%), White and Black African (1.30%), and White and Black Caribbean (2.50%).

^b^ This group is made up of Gypsy or Irish Traveller (2.20%), Roma (0.50%), White English or Irish (84.50%) and any Other White Background (12.80%).

Differences between groups in continuous variables were compared using independent samples t tests. As the cisgender group frequency distribution for both continuous variables were slightly negatively skewed, bootstrapping procedure stratified by the binary gender groups (TGD cf. cisgender) with 5,000 resamples, was applied. The output of the t test is reported alongside bias-corrected accelerated 95% confidence intervals from the bootstrapping procedure. Missing data and seemingly facetious responses ( *N* = 16) were managed via pairwise deletion and proportions of missing data were 11.85% overall and ranged from 0.00% to 22.30% for individual variables.

## Results

### Proportion of the sample that identifies as TGD

Of the available data, 98.60% reported gender identities that were categorised as cisgender and 1.40% reported identities which were categorised as transgender (binary and non-binary inclusive). A proportion of the sample did not report on gender identity ( *N* = 55; 1.11%) or sex assigned at birth ( *N* = 770; 15.54%). See Table 2 for a further breakdown of gender identity where data were available.

**Table 2.** Proportion of Transgender and Cisgender Adolescents in the Sample.

Gender Groups	Frequency	Percentage
Groups Disaggregated by ASAB		
Cisgender Girl	2,198	53.20%
Cisgender Boy	1,875	45.40%
Transgender Girl	8	0.20%
Transgender Boy	19	0.50%
Nonbinary (Female ASAB)	22	0.50%
Nonbinary (Male ASAB)	9	0.20%
Total	4,131	100.00%
Missing	823	-
Groups Not Disaggregated by ASAB		
Cisgender	4,073	98.60%
Binary Transgender	27	0.70%
Nonbinary	32	0.80%
Total	4,132	100.00%
Missing	822	-

Note. This table provides the frequency of those in the sample who were categorised by different gender groups. ASAB = assigned sex at birth.

### Socio-demographic profile of the TGD group compared to the cisgender group

The socio-demographic breakdown between the cisgender and TGD group is presented within Table 1.

### Health variables compared between the TGD group and the cisgender group

The chi-square found that TGD young people were significantly more likely to report the presence of a physical or mental illness compared to cisgender young people (see Table 1). The independent samples t-test found significantly higher RCADS depression and anxiety symptom scores in the TGD group compared to the cisgender group with a large, standardised effect size (see Table 3).

**Table 3.** Bootstrapped Independent Samples t Test Results.

	TGD	Cisgender	*t*	df	*p*	BCa 95% CI	*d*
RCADS Depression Subscale							
*N*	44	3155	-4.64 *	** 43.69**	**<.001**	**-18.96, -7.69**	**-0.92**
Mean (SD)	64.13 (18.94)	50.84 (14.32)					
Median	65.62	48.54					
Range	29.84–98.20	29.84–109.84					
RCADS Anxiety Subscale							
*N*	44	3155	-4.47 *	**43.62**	**<.001**	**-18.83, -7.40**	**-0.94**
Mean (SD)	60.38 (19.22)	47.40 (13.81)					
Median	56.14	43.99					
Range	32.20–100.08	28.78–114.40					

Note. This table provides the output of the t test that investigated differences in anxiety and depression scores for TGD young people compared to cisgender young people. TGD = transgender and gender diverse people. *t* = independent samples t test statistic.

* Equality of variances not assumed based on Levene’s Test for Equality of Variances. df = degrees of freedom. *p* = two-sided significance values. BCa 95% CI = bias-corrected accelerate 95% confidence intervals, via the bootstrap. *d* = Cohen’s *d* standardised effect size.

## Discussion

This study presented epidemiological data on the proportion of participants, aged 12 to 15 years old within the Born in Bradford Study Age of Wonder Cohort, that were TGD compared to those who were cisgender. Of the total sample 1.40% were TGD. This is consistent with other population estimates internationally ( Chew *et al.*, 2020; White *et al.*, 2023), albeit lower than estimates when using more expansive definitions of gender diversity ( Zhang *et al.*, 2020). Census data for over 16-year-olds nationally (0.50%) and in county in which Bradford is situated (Yorkshire and Humber: 0.52%), which reported that one in 200 young people aged 16 and over identified as TGD ( ONS, 2023a). However, it should be noted that the ONS have stated their concerns that this estimate may be inflated as respondents may have misinterpreted the question regarding gender, making it difficult to determine accuracy ( ONS, 2021). In addition, 6.00% of respondents did not answer the gender identity in the 2021 Census, a proportion that may include some TGD people and therefore suggests the possibility of an underestimation also ( ONS, 2023a). The proportion of the population who identified with a binary transgender identity (0.70%) were similar to those who identified with a nonbinary identity (0.80%) in this sample, contrasting with the 2021 Census estimates of 0.20% identifying as binary transgender and 0.06% as non-binary ( ONS, 2023a).

Of those who identified as TGD within this sample, they were significantly more likely to have female assigned sex on their birth certificates (70.00%) compared to male assigned sex. This is similar to the documented increases in representation of this group based on assigned sex ( Chew *et al.*, 2020; Taylor *et al.*, 2024). This was not found in the United States of America across 16 states where there was a higher representation of male assigned sex young people ( Turban *et al.*, 2022). Importantly, the overall sex distribution of the full sample was consistent with that of the age-matched Bradford and England more broadly, suggesting that this pattern is unlikely to be attributable to sampling bias ( Pickavance et al., 2025).

Furthermore, TGD adolescents were more likely to report being from a White ethnicity-related background and to be less likely to practice a religion than their cisgender counterparts, which is consistent with some findings ( de Graaf *et al.*, 2019) but not others ( Chew *et al.*, 2020). The overall distribution of ethnicity groups in the sample was broadly consistent with that of the age-matched Bradford population (e.g., 37.30% Pakistani, 6.90% Other Asian, 35.40% White British), but not with the England population overall (13.30% Pakistani, 8.70% Other Asian, 68.90% White British). This reflects the higher representation of Pakistani and other minoritised ethnicity groups in Bradford, alongside other demographic differences ( ONS, 2023b; Pickavance et al., 2025). In contrast, the sample overall (1.39%) was less likely to endorse having no religion than both the adult population in Bradford (28.20%) and England (36.70%; ONS, 2023b).

TGD young people within this sample were also observed to have higher markers of deprivation on some proxy variables (i.e. access to free school meals) but not others (i.e. national deprivation indices). Findings suggesting higher deprivation is congruent with a US-bases study exploring multiple proxies for socio-economic deprivation in TGD adults ( Carpenter *et al.*, 2020). However, the mechanisms of the disparity in findings of this study by deprivation measures are unclear, as research highlights that free school meal eligibility correlates highly with other measures of socio-economic disadvantage ( Taylor, 2018). This may be an artefact of relative deprivation within the postcodes covered in the Bradford district and used to calculate national deprivation indices, compared to an individualistic measure of deprivation such as accessing free school meals for each individual participant. More research is needed to explore the relationship between localities, deprivation and other specific aspects of socioeconomic status and how they exist for TGD youth, however. Overall, the sample showed a trend towards higher levels of deprivation compared with age-matched populations in Bradford and England more broadly ( Pickavance *et al.*, 2025).

Of the total sample, 18.40% had special educational needs (SEN), which is broadly consistent with national estimates and statistics for Bradford regarding the proportion of young people with SEN ( Department for Education, 2024; Pickavance *et al.*, 2025). However, this study found that TGD youth (30.60%) were twice as likely to have SEN compared to cisgender youth (15.40%), exceeding the national and local average ( Department for Education, 2024; Pickavance *et al.*, 2025). No known study has explored this specific outcome previously in TGD adolescents. However, this higher prevalence may be linked to increased neurodivergence or other learning difficulties within TGD populations, prompting schools to support access to SEN support for this group. Indeed, previous research suggests a connection between gender diversity and neurodevelopmental differences ( Kallitsounaki & Williams, 2023). However, this study did not specifically investigate causal factors or the presence of neurodivergence, thus this is not possible to conclude based on this study and thus further research is needed in this area.

There were no significant differences by age group or school year for the TGD group compared to the cisgender group within this sample, showing no difference in those identifying as TGD or cisgender by gender group, similar to previous research ( Chew *et al.*, 2020). This may suggest a stable rate of gender diversity within those from ages 12 to 15 years within this cohort. The overall age distribution of the sample was broadly consistent with age-matched populations nationally and within Bradford ( Pickavance et al., 2025).

TGD young people were more likely to endorse the presence of a physical or mental illness (49.10%) compared to cisgender young people (12.60%). TGD young people also scored higher on self-reported symptoms of depression and anxiety compared to cisgender young people with large effect sizes ( *d* = 0.92-0.94). Higher prevalence of physical and mental health difficulties in TGD populations compared to cisgender populations is well documented in the literature ( Chew *et al.*, 2020; Klinger *et al.*, 2024). which is congruent with the current findings.

Study strengths include use of a population cohort dataset which may increase the possibility for generalisation of findings to the local population. The locality of the population is also diverse regarding ethnicity, which provides unique insights into the sociodemographic and health profile of a diverse population ( Shire *et al.*, 2024).

Limitations include: non-inclusion of those schools which did not consent to take part, relatively high levels of missing data (including some likely facetious identity descriptors); the use of “male” and “female” options for gender identity as opposed to “boy” or “girl”, which may have increased confusion between sex and gender identity; reduction of complex ethnicity groups and avoidance of splitting TGD and ethnic groups finely to avoid reidentification issues; the inclusion of physical and mental health in one question and not as separate questions, which would have provided more detail; and a relatively simple analysis strategy that limits drawing of causal relationships between TGD and health status.

In conclusion, this study explored the proportion of TGD young people in a an ethnically-diverse population cohort dataset of young people aged 12 to 15 years, revealing that around 1.40% were classified as TGD and that more female-assigned-sex young people identified as TGD than male-assigned-sex young people. This study also investigated the sociodemographic and health profile of the TGD group compared to the cisgender group. The findings indicate an association where TGD CYP were less likely (compared to cisgender peers) to be from a minoritised ethnicity-related group and to practice a religion and were more likely to have physical or mental health difficulties, special educational needs, and access to free school meals. TGD groups also scored significantly higher on a measure of anxiety and depression symptoms compared to cisgender young people. There were no significant differences between groups in age or national deprivation indices. Future research should focus on longitudinal studies to track the identity and health trajectories of TGD youth and evaluate the effectiveness of different intervention strategies to support with health and wellbeing.

## Consent

The Born in Bradford’s Age of Wonder Cohort data collection was provided ethical approval by the Bradford Leeds NHS Research Ethics Committee (Ref: 21/YH/0261; ethical approved provided on 22/12/2021), which ensured that this study adhered to the Declaration of Helsinki. For all measures used in this analysis, there were three steps: 1) Headteacher signed written consent forms and a data-sharing agreement on behalf of their school; 2) Parents/carers were informed that their child’s data were being used for research in BiB and would be made available anonymously to other researchers worldwide. Video explanations were available in English and Urdu, and each school created a bespoke plan to ensure clear communication including additional activities, e.g parents’ evenings. Opt-out written consent forms and information sheets were distributed to pupils to give to their parents/carers. The schools were responsible for being satisfied that parents/carers were well-informed and had sufficient opportunity to opt-out (normally over a two-week period); 3) On assessment day, researchers asked the young person’s verbal assent to take part. For the questionnaire measures used in this analysis, young people had full autonomy over answering any, all, or none of the questions.

## Data availability

Born in Bradford allows researchers to apply to access the study data through the Born in Bradford Executive Group. Researchers need to submit an Expressions of Interest (EOI) form to borninbradford@bthft.nhs.uk and the EOI will be reviewed at the monthly Born in Bradford Executive. More information about how to access Born in Bradford data can be found on the study website: https://borninbradford.nhs.uk/research/how-to-access-data/. The STROBE reporting guidelines checklist are available via OSF, checklist for article entitled “Socio-Demographic and Health Data of Adolescents Identifying as Transgender or Gender Diverse in the Born in Bradford’s Age of Wonder Cohort”, DOI: https://doi.org/10.17605/OSF.IO/67AHW. Questionnaires and example consent forms and information sheets are also available via:

Repository name: OSF,

Title of project: BiB Age of Wonder Gender Diversity Project Supplementary Materials

DOI: https://doi.org/10.17605/OSF.IO/67AHW. ( Camp (2025))

This project contains the following underlying data:

1. Age-of-Wonder-24-25-Questionnaire.pdf
2. AoW schools information sheet v2 11.02.22.pdf
3. AoW Year 8 parent PIS and consent opt out v3 13.01.22.pdf
4. STROBE Statement V1.0. 09.06.2025.pdf
5. STROBE Statement V1.0. 09.06.2025.pdf

Details of licence: CC-By Attribution 4.0 International

Permissions to share these materials via CC-By Attributional licence has been provided by the Born in Bradford team.
