# Supplementary material for: Socio-Demographic and Health Data of Adolescents Identifying as Transgender or Gender Diverse in the Born in Bradford’s Age of Wonder Cohort
Source: Wellcome Open Res. 2026 Jul 3;10:413. Originally published 2025 Aug 6. [Version 3] doi: 10.12688/wellcomeopenres.24147.3 (PMC13234551; doi:10.12688/wellcomeopenres.24147.3)
Supplement: Peer review history

# Reviewer report: Reviewer response for version 3

**DOI:** 10.21956/wellcomeopenres.29819.r159830

**Published:** 2026-07-07

**Authors:** Lauren Herlitz

**Affiliations:** 1 University College London, London, UK

I am happy with the changes that authors have made to this article.

Is the work clearly and accurately presented and does it cite the current literature?

Yes

If applicable, is the statistical analysis and its interpretation appropriate?

Yes

Are all the source data underlying the results available to ensure full reproducibility?

Yes

Is the study design appropriate and is the work technically sound?

Yes

Are the conclusions drawn adequately supported by the results?

Yes

Are sufficient details of methods and analysis provided to allow replication by others?

Yes

Reviewer Expertise:

Social policy, access to and delivery of health and education services for families with additional health needs.

I confirm that I have read this submission and believe that I have an appropriate level of expertise to confirm that it is of an acceptable scientific standard.

# Reviewer report: Reviewer response for version 2

**DOI:** 10.21956/wellcomeopenres.28602.r153993

**Published:** 2026-06-03

**Authors:** Lauren Herlitz

**Affiliations:** 1 University College London, London, UK

This is a well-written paper with the introduction, methods and results very clearly presented. 

 The discussion is concise, situating the findings within existing literature, and captures the limitations of the study. It is somewhat lacking in describing the implications of the study for research, policy or practice. Is this study about identifying the particular needs of TGD adolescents? If so, its a starting point, and it would be good for the authors to indicate where to go next with future research that also includes the views of YP and families (i.e. mixed methods). The inclusion of PPIE might have helped draw this out. 

Is the work clearly and accurately presented and does it cite the current literature?

Yes

If applicable, is the statistical analysis and its interpretation appropriate?

Yes

Are all the source data underlying the results available to ensure full reproducibility?

Yes

Is the study design appropriate and is the work technically sound?

Yes

Are the conclusions drawn adequately supported by the results?

Yes

Are sufficient details of methods and analysis provided to allow replication by others?

Yes

Reviewer Expertise:

Social policy, access to and delivery of health and education services for families with additional health needs.

I confirm that I have read this submission and believe that I have an appropriate level of expertise to confirm that it is of an acceptable scientific standard.

# Reviewer report: Reviewer response for version 2

**DOI:** 10.21956/wellcomeopenres.28602.r150882

**Published:** 2026-05-29

**Authors:** Emma Soneson

**Affiliations:** 1 University of Oxford, Oxford, England, UK

Many thanks for the opportunity to review this paper, which uses Born in Bradford Age of Wonder data to understand how many adolescents (aged 12-15y) are identifying as trans/gender diverse (TGD) in a diverse population in 2022/23 and to compare their sociodemographic profiles to those of their cisgender peers. Thanks also to Dr Knowles for providing a thorough review at the first stage, the suggestions of which I feel the authors have thoughtfully considered and incorporated into this version. I believe the changes have strengthened the manuscript.

 Overall, I think this manuscript brings new and important information to the field and will be happy to see it indexed. Whilst there are some limitations in the study’s design (e.g. that the question on gender uses terminology associated with sex; that mental and physical health conditions are combined into a single question), I felt these were transparently reported and discussed. I have some further comments and suggestions that I hope the authors will find useful in terms of adding clarity and rigour.

**Measurement, analysis and reporting of gender and sex**

1. It was not clear to me how the authors considered data from participants who selected ‘wish to enter own response’ data for the question regarding sex assigned at birth. They are not mentioned in the analysis section and are absent from Table 1 – were they treated as missing?
2. Given quite high numbers not describing their sex (n = 81) or gender (n = 38), I was wondering whether the authors might consider including these as an explicit category within a sensitivity analysis. There is an argument to be made (as the authors later reference in their discussion of ONS estimates) that perhaps these groups contain a disproportionate representation of TGD individuals, so an analysis includes them could be valuable.
3. The authors state *‘Self-described gender identities were merged into these categories where possible (N = 15) and treated as missing data where not possible (N = 16).’ *(p. 7). It wasn’t clear to me why half of the participants who described their own gender identity were counted as missing data. What did these 16 answers look like? For example, was there not enough information given to classify them accurately? Were they perceived to be disingenuous answers? Additional detail would be helpful here.
4. The RCADS-25 scoring guide uses age- and sex-standardised scores (there is not as yet specific guidance for TGD young people). It seems the authors have chosen to use sex assigned at birth as opposed to gender. Some comment on this might be useful, especially as the developers seek to make more inclusive scoring guidelines with relation to TGD identities (https://www.corc.uk.net/news-blogs/2025/04/developments-in-rcads-scoring-combined-gender-option/).

**Clarity of findings presentation**

1. In the Plain English Summary, I am worried about the line *‘Finally, transgender and gender diverse young people reported higher physical and mental health concerns compared to cisgender young people.’* (p. 2). I know this was written for simplicity, but I would hesitate to report it this way as it looks like a definitive statement that TGD young people have higher physical concerns AND higher mental health concerns, which is not possible to conclude from the data (due to this being presented as a combined item).
2. In general, it would be helpful to have additional text in the Results section to draw out the main points from the tables. Right now there is very little explanatory text, and so the onus sits with the reader to draw conclusions based on the tables.
3. It would similarly be helpful to have a numerator and denominator reported for every percentage given. For example, it is not readily apparent where the 1.40% figure comes from (particularly given that it is 1.50% in Table 2).
4. Whilst I really appreciate that the authors have added information on missingness, I found the way that this was presented in Table 1 to be confusing. Even after reading the table notes, I was not sure what was meant by three categories of ‘missing (uncategorised)’, ‘missing: gender not reported’, and ‘missing generally’, which makes it difficult to contextualise missingness and understand its potential implications for the findings.
5. Relatedly, I found it difficult to distinguish between types of missingness. Thinking of the gender and sex questions, I could not work out the differences in reporting in terms of who (1) did not participate in the survey at all, (2) participated in the survey but left questions blank, and (3) participated in the survey and chose ‘do not want to answer’. This is particularly important for sex assigned at birth, which has a very high reported missingness (~15%), especially compared with the analogous ~1% figure for gender. I also found this incongruence quite strange – why were 770 not willing to report sex, whilst only 55 were not willing to report gender?
6. There seem to me to be slight inconsistencies across reporting. From my reading, there should be 59 TGD participants (based on the Year Group variable in Table 1, which had no missing data). However, in Table 1, only 57 participants are accounted for in terms of sex on birth certificate (a question which, according to the Methods, was used to derive TGD identity). In Table 2, the disaggregated groups represent 58 TGD participants and the aggregated ones 59. There may be reasons for these discrepancies, but they are not readily apparent. I wonder whether they might come from individuals who did not report sex assigned at birth but who used free-text to indicate a TGD identity – if this is the case, it should be stated.

**Discussion**

 In general, I believe greater context and attention to detail could help to improve the utility and impact of the Discussion. Also, given vast differences in sampling and measurement, I would strongly recommend being explicit for all referenced studies about both (1) how TGD was defined/operationalised and (2) which age range was included. Specific examples of places where further clarification is needed include:

1. In the first paragraph, the authors state, *‘Census data for over 16-year-olds nationally (0.50%) and in county in which Bradford is situated (Yorkshire and Humber: 0.52%), which reported that one in 200 young people aged 16 and over identified as TGD (ONS, 2023a).’* (p. 10). In looking at that reference, I could not find any section focused on ‘young people aged 16 and over’ – is this meant to say *people *aged 16 and over? Furthermore, in all of the statistics that follow, do those pertain to the county, or are they national estimates?
2. In the second paragraph, the authors write *‘Of those who identified as TGD within this sample, they were significantly more likely to have female assigned sex on their birth certificates (70.00%) compared to male assigned sex.’* (p. 10). This makes it seem as though it is *70% more likely *for TGD young people to be AMAB, which I don’t think the authors meant to say. Similarly, the authors state that their findings are *‘similar to the documented increases in representation of this group based on assigned sex’*. (p. 10). This reads as though the authors’ findings suggest an increase, but as these are cross-sectional data, I would re-word this to avoid misinterpretation (particularly as the BiB study *does *have longitudinal data).
3. In the discussion of sample representativeness, is the figure for those with no religion (stated as 1.39%) definitely correct? My reading of Table 1 is that n = 761 did not report a religion and 3,536 did ( = 17.8% no religion).

Finally, I thought that the Discussion was fairly surface-level, focusing more on comparisons with other literature than on what the findings might mean for practice. Even though they are descriptive in nature, I believe these findings offer important insights and would be interested to see additional engagement with what they might mean for policy and practice. Given the expertise on the authorship team, I would be very keen to have more of their interpretations and suggestions for how these findings might be used to better support TGD young people.

Is the work clearly and accurately presented and does it cite the current literature?

Partly

If applicable, is the statistical analysis and its interpretation appropriate?

Yes

Are all the source data underlying the results available to ensure full reproducibility?

Yes

Is the study design appropriate and is the work technically sound?

Yes

Are the conclusions drawn adequately supported by the results?

Yes

Are sufficient details of methods and analysis provided to allow replication by others?

Partly

Reviewer Expertise:

Child and adolescent mental health

I confirm that I have read this submission and believe that I have an appropriate level of expertise to confirm that it is of an acceptable scientific standard, however I have significant reservations, as outlined above.

# Response

**Published:** 2026-06-16

**Authors:** Jake Camp

**Affiliations:** Department of Psychology, King's College London Institute of Psychiatry Psychology & Neuroscience, London, England, UK

We would like to thank the reviewer for their thoughtful and constructive comments. We found the feedback extremely helpful in improving the clarity, transparency, and interpretation of the manuscript. In response, we have clarified the handling of sex assigned at birth and gender identity data, expanded reporting of missing and unclassifiable responses, revised the presentation of prevalence estimates and subgroup classifications, corrected several areas of ambiguity and imprecision in the Results and Discussion sections, and expanded consideration of the implications for policy and practice. These revisions improve the transparency and interpretability of the manuscript while not altering the overall findings or conclusions. We address each comment in detail below.  

**1) Reviewer: It was not clear how the authors considered data from participants who selected ‘wish to enter own response’ for sex assigned at birth. Were they treated as missing?**

**Response: **Thank you for highlighting this point. We agree that the handling of participants who selected “wish to enter own response” for sex assigned at birth required greater clarification.   Twenty-three participants selected “wish to enter own response” for sex assigned at birth. During data cleaning, these responses were reviewed individually. Where sufficient information was available, responses were recoded into a sex assigned at birth category (17 recoded as male and 5 recoded as female). One response could not be confidently classified and was therefore treated as missing data in the cleaned analytic variable.   To improve transparency, we have added clarification to the Methods section describing how these responses were handled.**** Manuscript addition (Data Analysis section): “Participants who selected “wish to enter own response” for sex assigned at birth were reviewed during data cleaning and recoded into a sex assigned at birth category where sufficient information was available. Responses that could not be confidently classified were treated as missing data in the cleaned analytic variable.”  

**2) Reviewer: Given quite high numbers not describing their sex (n = 81) or gender (n = 38), would the authors consider including these as an explicit category within a sensitivity analysis?**

**Response:** Thank you for this suggestion. We considered whether participants with missing or unclassifiable sex assigned at birth and gender identity data should be included as a separate category in a sensitivity analysis. However, we decided against this approach because these cases did not represent a coherent analytic group. Rather, they reflected a heterogeneous mixture of item non-response, non-disclosure, ambiguous self-descriptions, and a small number of responses judged to be facetious during data cleaning.   As the primary aim of the study was to estimate the proportion and characteristics of participants who could be classified as cisgender or transgender/gender diverse, we considered it methodologically more appropriate to treat these responses as missing data rather than creating an artificial category with limited interpretability.   We agree, however, that exclusion of these cases may have led to some underestimation of the proportion of participants identifying as transgender or gender diverse. To acknowledge this possibility, we have clarified this point within the limitations section of the manuscript.   Manuscript addition (Limitations section): “In addition, some participants did not disclose or could not be confidently classified according to sex assigned at birth and/or gender identity. As these responses were treated as missing data, the proportion of participants classified as TGD may represent a slight underestimate of the true prevalence within the cohort.”  

**3) Reviewer: The authors state that self-described gender identities were merged where possible (N = 15) and treated as missing where not possible (N = 16). What did these 16 answers look like? Was there not enough information? Were they disingenuous?** **Response: **Thank you for highlighting this point. We agree that further clarification was warranted.   The self-described gender responses that could not be classified were predominantly responses that were not interpretable as gender identities or appeared non-serious. As a result, these responses could not be reliably classified into the analytic categories used in this study and were therefore treated as missing data. Self-described gender responses were only merged into analytic categories where the available information allowed reliable classification.   We have added clarification to the Methods section to make this process more transparent.**** Manuscript addition (Data Analysis section): Self-described gender identities were reviewed individually and merged into analytic categories where sufficient information was available for reliable classification. Responses were treated as missing where they were not interpretable as gender identities, appeared non-serious, or could not otherwise be classified with confidence.****

**4) Reviewer: The RCADS-25 uses age- and sex-standardised scores. The authors appear to have used sex assigned at birth rather than gender. Please comment, especially in light of newer inclusive scoring developments.**  

**Response:** Thank you for highlighting this important point. We agree that the scoring approach warrants explicit acknowledgement.   In the absence of validated gender-diverse normative reference groups for the RCADS-25, T-scores were calculated using the available age- and sex-standardised norms based on sex assigned at birth rather than gender identity. While this approach is consistent with current RCADS scoring guidance, we recognise that it may not fully reflect the experiences of some TGD young people. We also note that more recent RCADS scoring options include a combined gender category; however, this does not currently constitute a validated gender-diverse normative reference group.   We have added this as a limitation within the manuscript.   Manuscript addition (Limitations section): “RCADS-25 T-scores were calculated using age- and sex-standardised norms based on sex assigned at birth rather than gender identity. Although this follows current scoring guidance, it may not fully reflect the experiences of some TGD young people.”   

**5) Reviewer: In the Plain English Summary, the line about higher physical and mental health concerns is too strong because the item was combined.**  

**Response:** Thank you for highlighting this issue. We agree that the original wording could be interpreted as implying that physical and mental health conditions were assessed separately, whereas the study used a combined physical-or-mental-health item alongside separate measures of anxiety and depression symptoms. We have therefore revised the Plain Language Summary to more accurately reflect the measures used and the findings obtained.   Manuscript change (Plain Language Summary): “Finally, transgender and gender diverse young people were more likely than cisgender young people to report having a physical or mental health condition or illness on the combined health question used in this study, and they also reported higher levels of anxiety and depression symptoms on the questionnaire measure used.”  

**6) Reviewer: The Results section would benefit from more explanatory text.** **Response:** Thank you for this suggestion. We agree that additional explanatory text would improve the readability of the Results section. We have therefore added brief narrative summaries to each Results subsection to highlight the main findings and aid interpretation alongside the tabulated results.   Manuscript change (Results section): “Of participants with classifiable gender data, 4,073 of 4,132 (98.57%) were categorised as cisgender and 59 of 4,132 (1.43%) were categorised as transgender or gender diverse (binary and non-binary inclusive).”   “The TGD group differed significantly from the cisgender group across several socio-demographic variables. Compared with cisgender participants, TGD participants were more likely to be White, report having no religion, be eligible for free school meals, and have special educational needs. There were no significant group differences in age, school year, country of birth, or national deprivation quartile.”   

**7) Reviewer: Please report a numerator and denominator for every percentage. The 1.40% figure is not easy to derive, especially given 1.50% in Table 2.**

**Response: **Thank you for highlighting rounding artefacts in the results.   The core prevalence figure is:

- TGD = 59
- Classifiable total = 4,132
- Therefore: 59 / 4,132 = 1.43%, which rounds to 1.4%

  That will be reported as 59/4,132 (1.43%, rounded to 1.4%). The reason Table 2 appears to give 1.5% is that it shows 0.7% binary transgender and 0.8% non-binary, which are separately rounded values:

- 27 / 4,132 = 0.65% rounded to 0.7%
- 32 / 4,132 = 0.77% rounded to 0.8%
- 0.65% + 0.77% = 1.42%, but after rounding separately it appears as 1.5%

  Response: Response: We have corrected this rounding artefact by reporting percentages to two decimal places within Table 2 and have added numerators and denominators to the Results section when describing the overall prevalence estimate.   Manuscript changes: Results: “Of participants with classifiable gender data, 4,073 of 4,132 (98.57%) were categorised as cisgender and 59 of 4,132 (1.43%) were categorised as transgender or gender diverse (binary and non-binary inclusive).”   Corrected within Table 2.   Discussion: “Of the total sample 1.43% were TGD. This is consistent with other population estimates internationally ( Chew et al., 2020; White et al., 2023), albeit lower than estimates when using more expansive definitions of gender diversity ( Zhang et al., 2020)”   “The proportion of the population who identified with a binary transgender identity (0.65%) was similar to those who identified with a nonbinary identity (0.77%) in this sample, contrasting with the 2021 Census estimates of 0.20% identifying as binary transgender and 0.06% as non-binary (ONS, 2023a).”   “Among participants classified as TGD and with available sex assigned at birth data, 40 of 57 (70.18%) had been assigned female sex at birth and 17 of 57 (29.82%) had been assigned male sex at birth, indicating a higher representation of AFAB than AMAB participants within this subgroup.”   “In conclusion, this study explored the proportion of TGD young people in a an ethnically-diverse population cohort dataset of young people aged 12 to 15 years, revealing that 1.43% were classified as TGD and that more female-assigned-sex young people identified as TGD than male-assigned-sex young people.”  

**8) Reviewer: The missingness presentation in Table 1 is confusing (“missing uncategorised”, “missing: gender not reported”, “missing generally”).**

**Response:** Thank you for highlighting this issue. We agree that the original labels did not clearly distinguish between different sources of missingness and may therefore have been difficult to interpret. To improve clarity, the table headings have been revised to distinguish participants whose gender identity data could not be classified from those with missing data for the variable of interest.   Manuscript change Table 1 column headings were changed to:

- Cisgender
- TGD
- Gender group not classifiable
- Variable missing
- χ²

 

**9) Reviewer: It is difficult to distinguish between not participating, leaving items blank, and choosing “do not wish to answer”, especially for sex assigned at birth. Why were 770 missing sex versus only 55 missing gender?**

**Response:** Thank you for highlighting this issue. We agree that the distinction between item non-response, “do not wish to answer” responses, and missing values in the cleaned analytic variables was not sufficiently clear.   The number of participants recorded as having missing sex assigned at birth data reflects the cleaned analytic variable rather than active refusal to answer the question. Specifically, this category includes participants who left the item blank, participants who selected “do not wish to answer”, and a small number of self-described responses that could not be confidently classified during data cleaning.   Similarly, missingness for gender identity reflects a combination of item non-response, “do not wish to answer” responses, and responses that could not be classified. The apparent discrepancy between the number of participants recorded as missing on the cleaned sex assigned at birth and gender identity variables therefore does not reflect active refusal alone.   To improve transparency, we have clarified this distinction within the manuscript. Manuscript addition (Data Analysis section): “Missing values in the cleaned sex assigned at birth and gender identity variables reflected a combination of item non-response, “do not wish to answer” responses, and responses that could not be confidently classified during data cleaning.”  

**10) Reviewer: There seem to be inconsistencies across reporting: 59 TGD in one place, 57 in Table 1 sex-on-birth-certificate row, 58 in Table 2 disaggregated groups, 59 in aggregated groups.**  

**Response:** Thank you for highlighting this issue. The differing totals reflect the use of different analytic denominators rather than inconsistencies in the data.   Fifty-nine participants were classified as TGD in the aggregate gender-group variable. One of these participants could not be allocated to an assigned sex at birth (ASAB)-specific subgroup, resulting in a total of 58 participants in the ASAB-disaggregated breakdown. In addition, two participants classified as TGD had missing or unclassifiable sex assigned at birth data and were therefore not included in the sex-on-birth-certificate analysis, resulting in a total of 57 participants for that specific row.   To improve transparency, explanatory footnotes have been added to the relevant tables.   Manuscript addition: Foot note for Table 1: The aggregate analytic gender-group variable classified 59/4,132 participants as TGD. The lower TGD total in the sex on birth certificate row (57) reflects row-specific missing/unclassifiable sex data for two participants who were classified as TGD in the aggregate variable. Percentages for cisgender and TGD groups are calculated from available data for each row.   Foot note for Table 2:  In the aggregate gender-group classification, 59/4,132 participants were classified as TGD. In the ASAB-disaggregated breakdown, TGD subgroups sum to 58 because one participant classified as TGD in the aggregate variable could not be allocated to an assigned-sex-at-birth (ASAB)-specific subgroup. Thus, the aggregated and ASAB-disaggregated totals use slightly different effective denominators for subgroup allocation. Percentages are based on participants with sufficient data for the relevant classification. ASAB = assigned sex at birth.  

**11) Reviewer: state more clearly how TGD was defined and what age range the comparison studies used.**

**Response:** Thank you for this suggestion. We agree that comparisons of prevalence estimates should be interpreted in light of differences in both the age ranges sampled and the operationalisation of transgender and gender diverse (TGD) status across studies. We have therefore added clarification to the Discussion noting that the comparison studies include adolescent, young adult, adult, and clinical referral samples, and that TGD status has been defined in different ways across studies, including direct self-identification, discordance between gender identity and sex assigned at birth, referral to specialist gender services, and broader measures of gender diversity.**** Manuscript addition (Discussion section): “When comparing these estimates, it is important to note that prevalence varies according to both the age range sampled and how transgender/gender minority status is operationalised. The studies cited here include children, adolescent, young people, and clinical referral samples, and have defined transgender/gender minority status using direct self-identification, discordance between gender identity and sex assigned at birth, referral to specialist gender services, or broader measures of gender diversity.”  

**12) Reviewer: The sentence about ONS Census data says “young people aged 16 and over”; is this meant to be people aged 16 and over? Are those regional or national statistics?** **Answer**  

**Response:** Thank you for highlighting this ambiguity. We have clarified that these estimates relate to people aged 16 years and over and have specified the national and regional (Yorkshire and Humber) Census estimates. We have also added text noting that these figures are not directly comparable with the present study because they relate to an older age range and use a different measurement approach.   Manuscript addition (Discussion section): “By comparison, Census 2021 data for people aged 16 years and over indicated that 0.50% of usual residents nationally, and 0.52% of residents in Yorkshire and Humber, reported that their gender identity differed from their sex registered at birth. However, these estimates should be interpreted cautiously given subsequent concerns about possible misunderstanding of the census question in some groups (ONS, 2021). Furthermore, these estimates are not directly comparable with the present study because they relate to an older age range and use a different measurement approach.”  

**13) Reviewer:** **The sentence saying TGD participants were “70% more likely” to have female assigned sex at birth is misleading, and saying findings are similar to “documented increases” sounds longitudinal.**  

**Response:** Thank you for highlighting this issue. We agree that the original wording could be interpreted as implying a relative increase or longitudinal trend. We have therefore revised the text to describe the observed sex assigned at birth distribution within the TGD subgroup more precisely and to avoid language suggesting temporal change. Specifically, we now report that approximately 70% of TGD participants with available sex assigned at birth data were assigned female sex at birth and describe this as a higher representation of AFAB than AMAB participants within the subgroup. We have also revised the comparison with previous literature to refer to a higher representation of AFAB participants in TGD samples, rather than "documented increases".   Manuscript changes (Discussion section): “Among participants classified as TGD and with available sex assigned at birth data, 40 of 57 (70.18%) had been assigned female sex at birth and 17 of 57 (29.82%) had been assigned male sex at birth, indicating a higher representation of AFAB than AMAB participants within this subgroup. This pattern is consistent with recent clinical and non-clinical adolescent studies that have reported a higher representation of individuals assigned female sex at birth within TGD samples (Chew et al., 2020; Taylor et al., 2024). However, this was not found in a population-based study of adolescents across 16 US states, where there was a higher representation of AMAB than AFAB young people within the TGD group (Turban et al., 2022).”   “In conclusion, this study explored the proportion of TGD young people in a an ethnically-diverse population cohort dataset of young people aged 12 to 15 years, revealing that 1.43% were classified as TGD and that more individuals assigned female sex at birth were more highly represented than those assigned male sex at birth within the TGD subgroup.”    

**14) Reviewer: Is the “1.39% no religion” figure correct? Table 1 suggests 17.8%.** **Response: **Thank you for noticing this error. The reported value of 1.39% was incorrect and reflected a prevalence estimate from elsewhere in the manuscript rather than the proportion reporting no religion. We have corrected the text accordingly.   Manuscript change (Discussion section): “In contrast, the sample overall was less likely to report having no religion than both the adult population in Bradford (28.2%) and England (36.7%; ONS, 2023b).”****

**15) Reviewer: The Discussion is too surface-level and should say more about what the findings mean for policy and practice.**  

**Response:** Thank you for this suggestion. We agree that the original Discussion did not sufficiently consider the implications of the findings for practice and policy. We have therefore expanded the Discussion to consider the potential implications for mental health service provision, recognition of intersecting educational and socioeconomic vulnerabilities, and the collection of sex assigned at birth and gender identity data within population studies and routine services.   Manuscript addition (Discussion section): “Although descriptive, these findings have several implications for practice and policy. First, the elevated burden of anxiety and depression symptoms, alongside higher rates of reported physical or mental health conditions among TGD young people, suggests the importance of ensuring timely access to appropriate mental health support within both educational and healthcare settings. Second, the higher representation of TGD young people among those eligible for free school meals and those with special educational needs highlights the importance of considering intersecting social, educational, and health-related vulnerabilities when planning support and allocating resources. Third, the challenges associated with missing, unclassifiable, and self-described sex and gender responses underscore the need for clearer and more inclusive approaches to collecting sex assigned at birth and gender identity data within population studies and routine service datasets. Improved data collection may support more accurate prevalence estimates and facilitate service planning for TGD young people.”  

# Reviewer report: Reviewer response for version 2

**DOI:** 10.21956/wellcomeopenres.28602.r148542

**Published:** 2026-02-24

**Authors:** Chris Knowles

**Affiliations:** 1 University of Manchester, Manchester, UK

Authors have responded to all my suggestions in a comprehensive and timely manner. I have no further comments and wish the authors all the best with their future work.

Is the work clearly and accurately presented and does it cite the current literature?

Yes

If applicable, is the statistical analysis and its interpretation appropriate?

Partly

Are all the source data underlying the results available to ensure full reproducibility?

Yes

Is the study design appropriate and is the work technically sound?

Yes

Are the conclusions drawn adequately supported by the results?

Yes

Are sufficient details of methods and analysis provided to allow replication by others?

Partly

Reviewer Expertise:

Adolescent wellbeing

I confirm that I have read this submission and believe that I have an appropriate level of expertise to confirm that it is of an acceptable scientific standard.

# Reviewer report: Reviewer response for version 1

**DOI:** 10.21956/wellcomeopenres.26638.r132947

**Published:** 2025-10-03

**Authors:** Chris Knowles

**Affiliations:** 1 University of Manchester, Manchester, UK

The article presents descriptive information on young people enrolled in the Born in Bradford study with particular emphasis on the sociodemographic and health profiles of those identifying as transgender and gender diverse (TGD) to better understand the proportion of young people in society who identify as TGD and ultimately, adapt and improve service provision to better support at-risk youth. The article is on the whole, comprehensive and easy to interpret. I have a few minor comments that I feel would improve the accuracy and clarity of writing.

 Introduction:

 1. The introduction provides strong rationale for the study and clearly articulates the contribution these prevalence statistics can make to the existing literature.

 Method/Procedures:

 2. While you provide a helpful reference for the data collection procedure, I think a brief overview herein would also be helpful to save the reader having to look elsewhere for the information.

 3. Please note the Year (and if possible, time of year) data were collected. I might have missed it, but I can’t see it reported anywhere in your manuscript. Please also report this in the abstract.

 Method/Measures:

 4. I’m a little confused as to why there was an option to self-describe in response to the question, “What is your sex? [The biological sex on your birth certificate]”. The premise of the item appears to be that biological sex is binary thus, can only be categorised as male or female. While I recognise that response options such as “do not wish to answer” or “don’t know” are valuable for reinforcing that participation is voluntary (which is particularly important given the sensitivity of the topic), it is less clear why a self-description option is necessary for this question. Might this not increase the likelihood of misclassification in an area where measurement error should, in principle, be minimal?

 5. By contrast, providing a self-description option for the gender question seems more appropriate and does not raise concerns. However, male and female are, generally speaking, terms used to denote biological sex. For the follow-up question on gender, the use of “boy/girl” as response options may have helped to draw a clearer distinction between sex and gender, both for respondents and readers. While I appreciate there’s not much can be done about it now, I would encourage authors to visit the possibility of changing the wording of the survey item pertaining to gender to refer to boy/girl if they deem it appropriate.

 6. Was the item on physical/mental illness phrased as a single combined question, or were these assessed separately? From a data precision standpoint, it would be preferable for these to be posed as distinct items, particularly given the elevated risk of mental as opposed to physical health difficulties among TGD young people. If they were in fact separate, it would be helpful to clarify this in the manuscript.

 7. While there are many possible reasons why young people may choose not to answer gender/sex-related questions, it is plausible that a proportion of missingness is likely attributable to participant uncertainty about identity and/or reluctance to disclose due to stigma. For this reason, it would be valuable to report the proportion of participants with missing data on either sex or gender as the percentage of total responses either in-text or alongside n's in Table 1. Doing so would provide useful context regarding the extent to which prevalence estimates may underestimate the true proportion of TGD youth.

 8. Was your measure of FSM actually FSM6 (i.e., whether pupils were ever eligible for free school meals over the past 6 six years) as per DfEs approach to measurement? See https://explore-education-statistics.service.gov.uk/find-statistics/school-pupils-and-their-characteristics/2024-25

 If so, please clarify.

 9. Table 1 is well laid out and easy to follow. However, given the focus on drawing comparisons between TGD and cisgender young people, it would be advantageous if percentages indicated the proportion of cis vs TGD within each sub-grouping. For example, amend the age section to:

            Cisgender,       TGD,           Missing

 age 12 680 ( **98.41%**), 11 ( **1.59%**), 179 ( **20.57%**)

 age 13 1400 ( **98.59%**), 20 ( **1.41%**), 273 ( **16.13%**)

 age 14 1444 ( **98.84%**), 17 ( **1.16%)**, 263 ( **15.26%**)

 age 15 549 ( **98.04%**), 11 ( **1.96%**), 106 ( **15.92%**)

 Note: missing %'s represent the proportion missing of overall sample (cis + TGD + missing), while other %'s represent proportion of available data. A footnote clarifying this point would also be useful.

 10. It would be helpful to clarify the rationale and process used to derive the ethnicity groupings presented in Table 1. Group A (“Asian, Black and Mixed groups”) appears to include "Roma" and "Gypsy or Irish Traveller" categories, both of which are classified under the broader “White” category in the 2021 Census (see: https://www.ethnicity-facts-figures.service.gov.uk/style-guide/ethnic-groups/). Why were they not listed under White?

 11. Expanding on this point, I would encourage the authors to re-think and re-group these ethnicities, if possible, to disaggregate White British from other minority White ethnicities. This suggestion is informed by recent evidence from a similar large-scale cohort study operating in northern England, which found that compared with White British young people, 10 of 16 minoritised ethnic groups (including White Irish and White Other) reported significantly higher wellbeing, while no minority ethnic groups reported significantly worse wellbeing (see: Reference 1). Distinguishing Pakistani as a stand-alone category is justified, given the regional significance and potential strategic advantages of addressing the needs of this comparatively large yet underserved group (see also, associations with bullying victimisation from the above study).

 Results:

 12. Inconsistent use of male/female, boy/girl. Please use consistent language throughout or provide a few explanatory sentences on why they are described as male/female in the methods and item wording but boy/girl in Table 2. See again my previous comment about suggested amend to question wording).

 13. Please use the term “young people” rather than simply “people”.

 Discussion:

 14. You note a couple of instances where your sample deviates from the national picture (number identifying as TGD, SEN). This is fine as you don’t claim to be nationally representative and regional variation is expected. It would however, be beneficial to include a table showing how your sample compares to *regional* averages on a few key metrics (Sex, SEN, FSM, and ethnicity data are all openly available) to offer greater transparency on the generalisability of your sample to the area from which it is selected.

Is the work clearly and accurately presented and does it cite the current literature?

Yes

If applicable, is the statistical analysis and its interpretation appropriate?

Partly

Are all the source data underlying the results available to ensure full reproducibility?

Yes

Is the study design appropriate and is the work technically sound?

Yes

Are the conclusions drawn adequately supported by the results?

Yes

Are sufficient details of methods and analysis provided to allow replication by others?

Partly

Reviewer Expertise:

Adolescent wellbeing

I confirm that I have read this submission and believe that I have an appropriate level of expertise to confirm that it is of an acceptable scientific standard, however I have significant reservations, as outlined above.

## References

[1] : Ethnic inequalities in adolescent mental wellbeing: An interaction analysis of social identity markers, risk and protective factors. *SSM - Mental Health* .2025;8: 10.1016/j.ssmmh.2025.100535 10.1016/j.ssmmh.2025.100535

# Response

**Published:** 2026-01-30

**Authors:** Jake Camp

**Affiliations:** Department of Psychology, King's College London Institute of Psychiatry Psychology & Neuroscience, London, England, UK

Thank you for your considered review. Please see below the response and ammendments we made to the second version. Introduction:

 1. The introduction provides strong rationale for the study and clearly articulates the contribution these prevalence statistics can make to the existing literature.  

**Authors Response:** thank you for this feedback.

 Method/Procedures:

 2. While you provide a helpful reference for the data collection procedure, I think a brief overview herein would also be helpful to save the reader having to look elsewhere for the information.

**Authors Response:** thank you for this suggestion. We have included further details in the methods section, for example: “ ***Sample***  Within the Age of Wonder dataset there were 4,954 participants aged 12 to 15 years (see Figure 1 for sample attrition). Mainstream schools within the Bradford District were approached to participate in the data collection. Participants were eligible to participate if, at the time of recruitment, they were: attending a school within the Bradford District that consented to take part; were in school years 8 to 10; the young person assented to take part; and that the schools were able to contact parents/carers with information about the study and then did not opt out. Participants were not eligible to take part if the parent/carer opted out, the participant did not assent, or a teacher deemed it inappropriate for them to participate. ***Procedures*** An in-depth description of the procedures employed to collect the data are described in Shire et al. (2024). Online questionnaires were developed in consultation with experts, parents, and young people, and were completed via the REDCap online survey platform hosted by Bradford Teaching Hospital NHS Foundation Trust (Harris et al., 2019). Questionnaires were administered by school staff by sending a unique link to students’ email address or via tablet computers supervised by members of the research team. Schools also provided data from their records (more detail in the Measures section). Data was collected between September 2022 and July 2023.”

 3.Please note the Year (and if possible, time of year) data were collected. I might have missed it, but I can’t see it reported anywhere in your manuscript. Please also report this in the abstract.

**Authors Response:** We have now added into the methods section: “Data was collected between September 2022 and July 2023.”

 4. I’m a little confused as to why there was an option to self-describe in response to the question, “What is your sex? [The biological sex on your birth certificate]”. The premise of the item appears to be that biological sex is binary thus, can only be categorised as male or female. While I recognise that response options such as “do not wish to answer” or “don’t know” are valuable for reinforcing that participation is voluntary (which is particularly important given the sensitivity of the topic), it is less clear why a self-description option is necessary for this question. Might this not increase the likelihood of misclassification in an area where measurement error should, in principle, be minimal?

**Authors Response:** thank you for this feedback. Questions were developed by the BiB team in consultation with experts, parents, and young people, which we have now mentioned within the manuscript (“Online questionnaires were developed in consultation with experts, parents, and young people, and were completed via the REDCap online survey… ^”^). The option to describe less common answers eg indeterminate/not-specified/VSC/DSD was felt to be useful. We will take this forward to the BiB AoW planning team to consider.

 5. By contrast, providing a self-description option for the gender question seems more appropriate and does not raise concerns. However, male and female are, generally speaking, terms used to denote biological sex. For the follow-up question on gender, the use of “boy/girl” as response options may have helped to draw a clearer distinction between sex and gender, both for respondents and readers. While I appreciate there’s not much can be done about it now, I would encourage authors to visit the possibility of changing the wording of the survey item pertaining to gender to refer to boy/girl if they deem it appropriate.

**Authors Response**: thank you for highlighting this. We will rediscuss this as a BiB team and included this in the limitations section: “the use of “male” and “female” options for gender identity as opposed to “boy” or “girl”, which may have increased confusion between sex and gender identity…“

 6. Was the item on physical/mental illness phrased as a single combined question, or were these assessed separately? From a data precision standpoint, it would be preferable for these to be posed as distinct items, particularly given the elevated risk of mental as opposed to physical health difficulties among TGD young people. If they were in fact separate, it would be helpful to clarify this in the manuscript.

**Authors Response**: yes, this was a single combined yes/no item “any physical or mental health conditions/illnesses”. We acknowledge the challenges you point out. We have now included this in the limitation section: “…the inclusion of physical and mental health in one question and not as separate questions, which would have provided more detail…”

 7. While there are many possible reasons why young people may choose not to answer gender/sex-related questions, it is plausible that a proportion of missingness is likely attributable to participant uncertainty about identity and/or reluctance to disclose due to stigma. For this reason, it would be valuable to report the proportion of participants with missing data on either sex or gender as the percentage of total responses either in-text or alongside n's in Table 1. Doing so would provide useful context regarding the extent to which prevalence estimates may underestimate the true proportion of TGD youth.

**Authors Response:** thank you for this suggestion. We have now included information on the proportion of data missing due to not answering questions on gender identity and sex assigned at birth in the results section (“A proportion of the sample did not report on gender identity ( *N* = 55; 1.11%) or sex assigned at birth ( *N* = 770; 15.54%)”) and included the breakdown of missing data due to not providing a gender identity in Table 2.

 8. Was your measure of FSM actually FSM6 (i.e., whether pupils were ever eligible for free school meals over the past 6 six years) as per DfEs approach to measurement? See https://explore-education-statistics.service.gov.uk/find-statistics/school-pupils-and-their-characteristics/2024-25

**Authors Response:** this was not FSM6 but instead was school-level data on whether participants had received free school meals in that academic year. This has been clarified in the methods section: “Of the data utilised in this study, schools provided data on the participants’ age and school year group, ethnicity, whether the participant had access to free school meals in that academic year, and their Special Educational Needs (SEN) status based on their records.”

 9. Table 1 is well laid out and easy to follow. However, given the focus on drawing comparisons between TGD and cisgender young people, it would be advantageous if percentages indicated the proportion of cis vs TGD within each sub-grouping. For example, amend the age section to:

            Cisgender,       TGD,           Missing

 age 12 680 ( **98.41%**), 11 ( **1.59%**), 179 ( **20.57%**)

 age 13 1400 ( **98.59%**), 20 ( **1.41%**), 273 ( **16.13%**)

 age 14 1444 ( **98.84%**), 17 ( **1.16%)**, 263 ( **15.26%**) age 15 549 ( **98.04%**), 11 ( **1.96%**), 106 ( **15.92%**) Note: missing %'s represent the proportion missing of overall sample (cis + TGD + missing), while other %'s represent proportion of available data. A footnote clarifying this point would also be useful.  

**Authors Response:** thank you for this suggestion. We have now changed the percentages in Table 2 as suggested and added a note to explain this in the table: “Note. % for TGD and cisgender young people represent the percentage for the available data, whereas % for those missing by gender not being reported and missing for other reasons represent the whole sample. TGD = transgender and gender diverse.”  

 10. It would be helpful to clarify the rationale and process used to derive the ethnicity groupings presented in Table 1. Group A (“Asian, Black and Mixed groups”) appears to include "Roma" and "Gypsy or Irish Traveller" categories, both of which are classified under the broader “White” category in the 2021 Census (see: https://www.ethnicity-facts-figures.service.gov.uk/style-guide/ethnic-groups/). Why were they not listed under White?

 11. Expanding on this point, I would encourage the authors to re-think and re-group these ethnicities, if possible, to disaggregate White British from other minority White ethnicities. This suggestion is informed by recent evidence from a similar large-scale cohort study operating in northern England, which found that compared with White British young people, 10 of 16 minoritised ethnic groups (including White Irish and White Other) reported significantly higher wellbeing, while no minority ethnic groups reported significantly worse wellbeing (see: Reference 1). Distinguishing Pakistani as a stand-alone category is justified, given the regional significance and potential strategic advantages of addressing the needs of this comparatively large yet underserved group (see also, associations with bullying victimisation from the above study).  

**Authors Response:** thank you for highlighting these two points. Unfortunately, any further disaggregation, including the any other white background group, results in at least one cell being *N *= <5, which is against the data sharing agreement and thus not possible to report. We have mentioned this in the limitation section: “…reduction of complex ethnicity groups and avoidance of splitting TGD and ethnic groups finely to avoid reidentification issues;” We have, however, moved the Roma and Gypsie participants into the White group.  

 Results:

 12. Inconsistent use of male/female, boy/girl. Please use consistent language throughout or provide a few explanatory sentences on why they are described as male/female in the methods and item wording but boy/girl in Table 2. See again my previous comment about suggested amend to question wording).

 13. Please use the term “young people” rather than simply “people”.

**Authors Response: **Thank you for highlighting these points. We have now made these consistent where accurate to do so.

 14. You note a couple of instances where your sample deviates from the national picture (number identifying as TGD, SEN). This is fine as you don’t claim to be nationally representative and regional variation is expected. It would however, be beneficial to include a table showing how your sample compares to *regional* averages on a few key metrics (Sex, SEN, FSM, and ethnicity data are all openly available) to offer greater transparency on the generalisability of your sample to the area from which it is selected.

**Authors Response:** Thank you for this suggestion. We agree that transparency regarding the comparability of the sample to the regional population is important. However, a recent publication has already provided a detailed comparison of a Born in Bradford Age of Wonder cohort with both regional and national demographic indicators, including sex, ethnicity, special educational needs, and socioeconomic deprivation (Pickavance et al., 2025). Given this, we did not replicate these comparisons in a separate table. Instead, we have integrated key comparative findings from this work, alongside the most recent Census data where relevant, directly into the text to contextualise our sample and support interpretation of generalisability.
